# Evaluation of mtDNA analysis as a screening method prior to individual identification by short tandem repeat analysis of sika deer (*Cervus nippon*) for illegal disposal of hunting in Japan

**DOI:** 10.3389/fvets.2025.1599909

**Published:** 2025-09-16

**Authors:** Aki Tanaka, Reina Ueda, Chihiro Udagawa, Toshinori Omi, Yuko Kihara, Shin-ichi Hayama

**Affiliations:** Department of Veterinary Medicine, Nippon Veterinary and Life Science University, Musashinoshi, Tokyo, Japan

**Keywords:** illegal disposal, MtDNA analysis, wildlife forensics, screening, individual identification

## Abstract

The sika deer (*Cervus nippon*) is subject to controlled abatements Japan, and in many areas hunters are subsidized by submitting tails from the dead deer. The carcasses must be properly disposed of after the tails are removed, and abandoning culled animals in the field is strictly prohibited by law. However, it has become an increasing legal problem that carcasses are left behind without proper disposal. In such cases, individual identification by DNA analysis has been considered useful to identify the culled animals and the suspects who abandoned the carcasses, and to provide scientific evidence for criminal investigations. In this study, the mtDNA D-loop region was analyzed in Sika deer using 285 deer samples with the aim of evaluating the capability of mtDNA markers as a screening method prior to performing individual identification by short tandem repeat analysis. Haplotype data obtained from 283 samples, excluding those with confirmed heteroplasmy, were used to calculate probability of random match, power to exclude, and genetic diversity. Twenty-three haplotypes were detected in 285 Japanese deer from the same local population, with mutations in the tandem repeat sequence and 48 different sites. The exclusion probability was 79.9%. The results suggested that mtDNA analysis provided moderate identification capability for screening. mtDNA analysis has proven to be a useful robust analysis in wildlife forensics when the samples were decayed and there were time and resource limitations, and is expected to be applied to solve illegal disposal of animal carcasses.

## Introduction

Wildlife forensics is the application of scientific disciplines to enforce laws and solve illegal problems involving wildlife, and consists of taxonomy, pathology, molecular biology, biochemistry, genetics and toxicology (1). In addition to evidence collection and the prosecution of wildlife crimes, veterinary forensic science contributes to a wide range of wildlife-related issues, such as monitoring environmental change and investigating epidemiology of disease (1). In many cases of suspected wildlife crime, unlike human cases, there is no “victim” to provide information for the investigation. Therefore, wildlife forensics technique play important role in wildlife crimes by connecting the suspect, victim and crime scene with trace and degraded physical evidence recovered from the crime scene (2).

Morphological, isotopic and DNA analyses are used to identify evidence (3). Of these, DNA evidence is particularly important for evidence of wildlife origin, which is often already degraded, decayed or morphologically unidentifiable (4). Development and application of DNA analysis techniques to provide evidence applicable in wildlife crime investigations, commonly referred to as ‘wildlife DNA identification’, has long been recognized, but is now gaining more attention as an important discipline (5).

Sika deer (*Cervus nippon*) is a large wild mammal widely distributed throughout East Asia (6). Its distribution in Japan has been fragmented by human activities: since the mid-19th century, habitat fragmentation, overhunting and several severe winters have led to the extinction of many local populations and a sharp decline in their numbers (7). After the Second World War, the ‘Hunting Law’ was amended into the ‘Law on the Protection of Birds and Animals and Hunting’ and the hunting of female deer was banned from 1948. Although the hunting ban was lifted in some areas in 1994, a policy of protection was in place until 2007. As a result, sika deer population increased, especially in northern and central mainland, and its range expanded rapidly (8, 9).

Various problems such as damage to forestry, agriculture and vegetation have been reported in various areas in Japan due to the increase in the sika deer population, and most prefectures are managing the populations based on Type II Specified Birds and Wild Animals Management Plans (10), [Ministry of the Environment: Results of Population Estimates of Japanese Deer and Wild Boar Nationwide (2023). https://www.env.go.jp/press/110760_00001.html confirmed on 12 June 2023]. To encourage such population management, subsidies are provided in many areas to those who hunted the sika deer by submitting the tail to the local government.

Culled animal carcasses must be handled properly, and it is forbidden to leave behind in the field. However, there have been increasing cases in Japan where culled animal carcasses had been abandoned without proper disposal and this has become a serious legal issue. Investigation of such cases, determining the genotypes of the tails submitted to the government at the time of capture and of the abandoned individuals, and investigating whether there are identical individuals that match, will provide beneficial scientific evidence for the crime investigation and lead to the deterrence of the crime. Individual identification through DNA analysis is therefore required and crucial to solve these cases.

mtDNA analysis has attracted attention in forensic science because of its potential to provide valuable results from samples for which complete and reliable nuclear DNA results cannot be obtained (11–13). In cats, mtDNA analysis has proven to be useful for individual identification when STR analysis was not possible (14). mtDNA can be isolated from skin, hair shaft, ivory, feathers, scales, horns, etc. (12, 13) and is widely used in wildlife forensics due to its feasibility of isolation (14). In addition, because many of the samples collected for wildlife forensics are highly degraded, the success rate of nuclear DNA amplification may be lower than mtDNA, making mtDNA potentially the only DNA analysis that can be used for individual identification (15, 16). The use of mtDNA in wildlife forensics is widespread because of its abundance in samples where nuclear DNA may be degraded and highly processed, and because of the reduced time required for method development (17). However, mtDNA alone has limitations for individual identification because it is maternally inherited, leading to a lack of differentiation between siblings or closely related individuals from the same maternal lineage (18). mtDNA is inherited maternally, which does not provide the same level of individual specificity as nuclear DNA, but could be used when samples are too degraded for short tandem repeat (STR) analysis (19).

The D-loop region, located within the regulatory region (CR) of mtDNA, is the largest non-coding part of the molecule and is prone to conserved base substitutions and shows a high rate of evolution (9). The D-loop region is the most appropriate region to screen for mutations for individual identification (20, 21). In humans, this region has been found to be highly polymorphic and is adopted in individual identification (21–23). In sika deer, the D-loop region of mtDNA has been used in several studies aimed at revealing the genetic structure of multiple local populations (7, 24–28). The D-loop region of sika deer mtDNA is approximately 1,100 bp (7), and many studies have used a portion of this region for analysis. However, analysis of the entire D-loop region may provide new insights into the genetic diversity of deer. In addition, the D-loop region of the sika deer contains a tandem repeat sequence consisting of approximately 37–40 bp of similar sequences (24, 29), and analysis of this region may provide additional information on the sika deer mtDNA D-loop region.

According to the guidelines for mtDNA typing recommended by the DNA Committee of the International Society of Legal Genetics, sequencing of the entire CR is necessary to obtain the most reliable results (11, 30). However, sample quality often makes it impossible to amplify the entire CR using just forward and reverse primers. In wildlife forensics, the quality of DNA extracted from decaying animals is often low, which makes amplification of large DNA fragments difficult. The ISFG guidelines state that reported mtDNA sequences should be based on multiple sequence data, including forward and reverse primers whenever possible (31). If sequencing with forward and reverse primers was not possible, multiple sequence data obtained from the same DNA strand with different primers should be used. Therefore, it may be possible to construct more reliable data on the entire mtDNA D-loop region of sika deer by sequencing from both directions using multiple primer.

If the mtDNA haplotypes of evidence samples and individual match in individual identification, the following could be considered: (1) they belong to the individual thought to have provided the evidence; (2) they belong to the same maternal lineage as the individual thought to have provided the evidence; or (3) the mtDNA of the evidence is a match to that of the individual thought to have provided the evidence. Because estimates of DNA mutation rates for mtDNA CR vary between species (32, 33), the discriminatory power of mtDNA CR may be higher or lower in different species. Studies evaluating such discriminatory power for the sika deer in Japan are scarce. Therefore, it is necessary to evaluate the discriminatory power of the mtDNA CRs of the sika deer before reporting. Since mtDNA haplotypes may match in many maternally related individuals, the discriminatory power depends not only on sequence diversity, but also on the size of the geographically related genetic database (34). Therefore, databases should be constructed for each regional population. However, there are no studies that have conducted mtDNA haplotype database construction with individual identification or evaluated individual identification ability by statistical analysis in sika deer.

When conducting DNA analysis of sika deer, one important factor to consider is the decline in genetic diversity. Sika deer have experienced a population bottleneck due to a drastic decrease in their numbers (35). A bottleneck occurs when a population undergoes a significant reduction in size due to factors such as overhunting, habitat loss caused by economic development, or habitat fragmentation, leading to reduced genetic diversity and lower levels of gene flow (35–38). Since bottlenecks diminish genetic diversity, it is crucial to account for their effects even if the current population size is large. Additionally, habitat fragmentation can lead to genetic differentiation among local populations (25, 26, 39), and previous studies have reported that the genetic structure of sika deer varies among regional populations (24, 35, 40).

The purpose of this study was to conduct DNA identification at the request of the law enforcement to determine the genotypes of tails of sika deer surrendered to the government and abandoned carcasses, and to evaluate as screening method prior to STR analysis for identical individuals that match.

DNA analysis in wildlife forensics requires the use of appropriate markers for the species under investigation. Since there are differences in genetic structure among regional populations, verification of the availability of markers for each assessment is inevitable. However, there are few studies in Japan that have verified the usefulness of markers and constructed databases for application to wildlife DNA analysis. Therefore, in this study, we analyzed the D-loop region, an mtDNA CR, with the aim of constructing a database of mtDNA haplotypes of sika deer in local population, and, at the same time, we evaluated the individual identification ability of this region and examined its application for wildlife forensics.

## Materials and methods

### Samples

A total of 283 tails and two lower jaw samples from a total of 285 sika deer were submitted to the university by the law enforcement in April 2022. Muscle fragments were collected with a scalpel and frozen at −30°C until DNA extraction.

### DNA extraction

DNA was extracted from 25 mg muscle pieces to a final yield of 100 μL using a DNA extraction kit (DNeasy Blood & Tissue Kit, Qiagen, Venlo, The Netherlands) according to protocol. NanoDrop Lite (Thermo Fisher Scientific Inc., Walthman, MA, USA) was used to measure DNA concentration and purity. The extraction products were stored at −20°C until polymerase chain reaction (PCR).

### Amplification of the sika deer mtDNA D-loop region by PCR

Until March 2022, PCR was performed following the GoTaq® Colorless Master Mix protocol (Promega, Madison, WI, USA), with a reaction mixture of 25 μL containing 2 × GoTaq® Colorless 1 × Master Mix, 0.4 μM of each primer, less than 250 ng of template DNA per 25 μL, and distilled water. The PCR conditions included an initial denaturation at 95°C for 15 min, followed by 32 cycles of denaturation at 95°C for 30 s, annealing at 55°C for 30 s, and extension at 72°C for 1 min, with a final extension at 72°C for 5 min.

From April 2023 onward, PCR was conducted using the KOD FX Neo polymerase (TOYOBO Co., Ltd., Osaka, Japan) with a reaction mixture of 25 μL containing 1 × PCR Buffer for KOD FX Neo, 0.4 mM dNTPs, 0.3 μM of each primer, less than 100 ng of template DNA per 25 μL, 1 U/25 μL KOD FX Neo, and distilled water. The PCR conditions consisted of an initial denaturation at 94°C for 2 min, followed by 30 cycles of denaturation at 98°C for 10 s, annealing at 57°C for 30 s, and extension at 68°C for 1 min.

PCR products were subjected to electrophoresis on a 1.5% agarose gel for 20–25 min (1 × TAE buffer [Promega, Madison, WI, USA], Loading Buffer [Nippon Gene, Japan]) at 100 V/cm using a Mupid-2plus electrophoresis system (TaKaRa Co., Ltd., Japan). After electrophoresis, the gel was stained with ethidium bromide (EtBr, Nippon Gene, Japan) for 15 min, and band amplification was confirmed under UV illumination.

### Sequencing by direct sequencing

The amplified PCR products were purified according to protocol using ExoSAP-IT Express (Thermo Fisher Scientific Inc., USA). The purified products were sequenced by direct sequencing by Eurofin Genomics Inc. Eight primers were used for direct sequencing, which were prepared according to the reports of Nagata and colleagues (7), Nabata and colleagues (41) and Yoshio and colleagues (25) (Table 1).

**Table 1:** Primers used for analysis of sika deer (*n* = 283) mtDNA D-loop region.

Primer	Sequence	Reference
L15926	5′-CTAATACACCAGTCTTGTAAACC-3′	Nagata et al. (1998) (7)
H597	5′-AGGCATTTTCAGTGCCTTGCTTTG-3′	Nagata et al. (1998) (7)
LD5	5′-AAGCCATAGCCCCACTATCAA-3’	Nagata et al. (1998) (7)
HD8	5′-TTGACTTAATGCGCTATGTA-3′	Nagata et al. (1998) (7)
HD2	5′-CCTGAAAAAGAACCAGATG-3′	Nagata et al. (1998) (7)
LD15	5′-TATATGCCCCATGCTTATAAGC-3′	Nagata et al. (2004) (41)
HD2R	5′-CATCTGGTTCTTTTTTCAGG-3′	Yoshio et al. (2008) (25)
HD6R	5′-GCAGTCAATGGTCACAGGAC-3′	Yoshio et al. (2008) (25)

The resulting sequences were multiple aligned using MEGA7.0 (42), and haplotypes were classified using GENETYX Ver. 12 (GENETYX Co., Tokyo, Japan).

### Statistical analysis

Haplotype data obtained from 283 samples, excluding those with confirmed heteroplasmy, were used to calculate the probability of random match, the power of exclusion, and the genetic diversity using the following formulae: The formula for the probability of random match was [P = ΣX_i_^2^], the formula for the power of exclusion was [PD = 1 -ΣX*_i_*^2^], and the formula for the genetic diversity was [h = n(1-ΣX*_i_*^2^)/ (n-1)]. (*i*: haplotype number, X*_i_*: *i*-th haplotype frequency, n: number of individuals) (43, 44).

### Ethics statement

Ethical approval for this study was obtained from Institutional Animal Care and Use Committee (approval number: 2024 K-29).

## Results

### PCR amplification of the D-loop region of sika deer mtDNA

In this study, PCR was performed on the D-loop region, a particularly highly polymorphic region of mtDNA, and PCR amplification yielded a clean single band at the expected position of approximately 1,000 to 1,200 bp (1,013 to 1,206 bp) for 283 sika deer samples (62).

Direct sequencing was then performed using the primers listed in Table 1 to determine the nucleotide sequence, and from all 285 samples a sequence of 1,013–1,206 bp including tandem repeat sequences was determined.

### Haplotype detection

Among the 1,013–1,206 bp sequenced in this study, mutations were found in the tandem repeat haplotype and 48 other sites, and 23 haplotypes were detected in the entire population (285 animals) (Table 2). The minimum number of mutation sites was one (haplotype b) and the maximum was 32 (haplotype n-2). Two samples showed heteroplasmy and were excluded from further analysis.

**Table 2:** Number of samples (*n*) and frequency of occurrence (%) of 22 haplotypes detected in 283 sika deer submitted in April 2022, Japan.

Haplotype	*n*	Frequency (%)
a	2	0.7
b	1	0.4
c-1	2	0.7
c-2	1	0.4
d	1	0.4
e	3	1.1
f	3	1.1
g	2	0.7
h-1	1	0.4
h-2	46	16.3
h-3	1	0.4
h-4	2	0.7
i	1	0.4
j	2	0.7
k-1	18	6.4
k-2	59	20.8
k-3	1	0.4
l-1	98	34.6
l-2	8	2.8
l-3	1	0.4
m	1	0.4
n-1	9	3.2
n-2	20	7.1
Total	283	100

The most predominant haplotype was haplotype l-1 with a frequency of 34.6% (n = 98). This was followed by haplotype k-2 (20.8%, n = 59) and haplotype h-2 (16.3%, n = 46). Fourteen haplotypes, including haplotype a and haplotype b, were rare haplotypes detected in only one to two samples. The mutation sites of the 22 haplotypes detected in 283 deer were summarized in Supplementary Table 1 (63).

In this study, tandem repeat sequences were detected from base 167 to base 516 and 11 tandem repeat haplotypes were detected (Table 3) (64). Five of these (TD-15, TD-4, TD-6, TD-2 and TD-9) were consistent with the tandem repeat haplotypes reported by Hata and colleagues (45). Six other types were newly discovered in this study. The number of tandem repeats ranged from four to nine.

**Table 3:** Eleven tandem repeat haplotypes detected in 283 two-tailed deer submitted in April 2022, Japan.

Tandem repeat haplotypes	Repeat units								
	1st	2nd	3rd	4th	5th	6th	7th	8th	9th
S-1	a1	d1	e1	n7	s1	s2	d8	d8	d8
TD-15	a1	d1	e1	n7	n13	d8	d8	d8	
S-2	a1	d1	n4	f3	n5	d8	s3	d8	
S-3	a1	d1	e1	n7	n13	n13	d8	d8	
S-4	a1	d1	e1	n7	n13	d8	d8		
TD-4	a1	d1	e1	n1	d8	d8	d8		
TD-6	a1	d1	e1	n1	d8	d8	n5		
S-5	a1	d1	n4	f3	d8	s4	d8		
S-6	a1	d1	n4	f3	d8	d8			
TD-2	a1	d1	e1	n1	d8	d8			
TD-9	n2	d5	n3	f4					

Nineteen repeat unit sequences were detected in this study. The list of repeat unit mutation sites was summarized in Supplementary Table 2: seven (a1, d1, d5, e1, d8, f3, f4) matched the sequences reported by Nagata and colleagues (24) and eight (n2, n4, n3, n1, n7, n10, n13, n5) matched those reported by Hata and colleagues (45). The other sequences were newly detected in this study.

NCBI BLAST search results did not reveal any complete haplotype matches. When partial sequence matches were examined, the partial sequence of haplotype c-1 and haplotype c-2 (1,019 bp) matched CN-4 (46), the partial sequence of haplotype h-3 (1,053 bp) matched CN-1 (46), and the partial sequence of haplotype h-4 (1,083 bp) matched Szo6 [Tamate unpublished].

### Statistical analysis

In this study, statistical analysis was performed using data from 283 samples, excluding 2 samples in which heteroplasmy was detected. The results showed that the random match probability was 20.1%, the exclusion power was 79.9% and the genetic diversity was 0.8016.

## Discussion

It has been reported that sika deer have reduced genetic diversity due to the effects of local bottlenecks (7, 25, 28). In many previous studies analyzing the sika deer mtDNA D-loop region, often performed excluding the tandem repeat region, but due to the effects of such bottlenecks, the number of mtDNA haplotypes detected in sika deer was relatively low, ranging from 4 to 13 (25, 26). In a previous study that included the tandem repeat region in its analysis, the number of haplotypes detected was 16 (45). However, in this study, a relatively large number of haplotypes (23 types) were detected. Possible reasons for this included the inclusion of the tandem repeat region in the analysis and regional differences in the effects of the population bottleneck.

In this study, 11 tandem repeat haplotypes were detected, and the number of tandem repeats ranged from four to nine. The number of tandem repeats differed between the southern and northern populations bordering Hyogo Prefecture, with the southern population having five or fewer repeats and the northern population having six or more repeats (24, 29). However, a mixture of haplotypes with characteristics of each of the southern and northern populations has been observed in the regional populations of the Kinki region (24), eastern Shikoku (29), southern Kanto (26) and Toyama Prefecture (47), and the results of this study were similar. Since the same local population was studied in this study, artificial introgression of individuals of same bloodline and resulting hybridization between the two populations were suggested. Although previous studies analyzing population structure have considered the number of tandem repeats, very few studies have gone as far as identifying tandem repeat haplotypes. In this study, tandem repeat haplotypes was identified and, similar to previous studies that have identified some tandem repeat haplotypes (24, 45), the tandem repeat region showed a particularly high mutation rate in the D-loop region. It was considered that this high mutation rate contributed to a more detailed classification of the haplotypes. In fact, excluding the tandem repeat sequences, the number of haplotypes in the populations studied in this study was 16. Therefore, when mtDNA analysis was used to identify individual sika deer, it was expected that the identification power would be increased by including tandem repeat haplotypes in the analysis.

The large number of haplotypes detected compared to previous studies suggests that the degree of reduction in genetic diversity due to bottlenecks depends on the extent and duration of bottlenecks in each local population. Particularly, in wild animals, gene flow can occur between neighboring local populations (26), which can reduce the impact of bottlenecks. Therefore, it was considered that when using mtDNA haplotypes to identify individuals, the mtDNA haplotype composition of each local population should be examined for each analysis, considering the locality to which the evidential sample belongs.

In this study, statistical analyses were conducted to evaluate the identification power of the sika deer by mtDNA analysis. The results showed that the random match probability was 20.1%, the exclusion power was 79.9%, and the genetic diversity was 0.8016. The exclusion capacity of 79.9% means that approximately 8 out of 10 randomly selected animals can be excluded as possibly being the same individual, and this dataset can be used to correctly exclude approximately 80 out of 100 animals unrelated to the forensic evidence sample as the source of the evidence sample.

In a previous study using haplotype diversity calculated with the same formula as the genetic diversity in this study, the haplotype diversity in a study examining the entire mtDNA genome of 287 deer from China, Russia and Japan was 0.9358 (48). The haplotype diversity in a study examining the mtDNA D-loop region of 171 deer was 0.916 for the southern population and 0.623 for the northern population, suggesting that the northern population has lower genetic diversity than the southern population (49). Although only one regional population was analyzed in this study, genetic diversity could be higher if several regional populations were analyzed together, as in the previous studies mentioned above. In this study, two haplotypes, haplotype n-1 and haplotype n-2, were characterized by the southern population and about 90% of the haplotypes were characterized by the northern population. The fact that many haplotypes with characteristics of northern populations with low genetic diversity were detected suggests that the value of genetic diversity was relatively low.

In previous study, the random match probability and haplotype diversity of Japanese mtDNA are 0.4% and 0.9969, respectively (22). For dogs and cats, where the usefulness of individual identification by mtDNA has been demonstrated, the random match probability and genetic diversity of canine mtDNA are 7.5% and 0.929, respectively (50), while the random match probability and genetic diversity of feline mtDNA are 14.1% and 0.9969, respectively (51). The probability and haplotype diversity of feline mtDNA are 14.1% and 0.8767, respectively (52). Both have a lower random match probability and higher genetic diversity compared to sika deer. The human mtDNA haplotype is thought to have diverged more than 150,000 years ago (27), the ancestors of domestic dogs diverged from wolves 100,000 years ago (53), and cats are thought to have diverged from five mitochondrial lineages that arose approximately 9,000 years ago (54). The probability of a random match and the level of genetic diversity are influenced by the divergence history of these mtDNA haplotypes. Compared with humans, dogs, and cats, a shorter divergence history corresponds to a higher probability of a random match and reduced genetic diversity. As many local populations of sika deer have been extinct since the mid-19th century (8), the increase in sika deer populations and subsequent increase in mtDNA haplotype divergence is likely to be more recent.

Compared to humans, dogs and cats, where mtDNA analysis has proven to be beneficial, the statistical values in this study indicated that it was not sufficient to correctly identify specimens in sika deer. However, they demonstrated sufficient utility as exclusionary evidence and, with the accumulation of further research data, may prove useful in the identification of individuals in the forensic veterinary study of sika deer. In addition to its use as a means of exclusion, this study proved that mtDNA analysis is a powerful tool for screening prior to performing STR analysis, especially when there is limitation in time and resource. In the past, a case was reported in which mtDNA and STR analysis were combined to develop a DNA test for individual identification of North Pacific minke whales sold in the Korean market (55).

DNA identification in wildlife forensics contributes to the identification of evidence in wildlife crimes and the prosecution of wildlife crimes, and genetic markers have now been developed and databases established to implement individual identification in various animal species. In Japan, most prefectures have implemented population control of sika under the Type II Specified Bird and Wildlife Management Plan due to the recent population increase, but there have been increasing cases where captured deer have been abandoned without proper disposal. This study was the first to evaluate the availability of mtDNA analysis for identification of sika deer in Japan and the results suggested that mtDNA analysis provided effective tool as screening and exclusion when there were large number of samples that needed to be analyzed within certain limit of time and resource, and when the samples were decayed, which is common in wildlife forensic cases. mtDNA analysis is proven to be beneficial in wildlife forensics and expected to be applied to solve legal problem involving wildlife such as illegal disposal of animal carcasses. Illegal disposal is a global issue involving not only domestic animals but also wildlife such as roe deer (*Capreolus capreolus*) and wild boars (*Sus scrofa*), often causing health concerns due to scavenging phenomena and the transmission of infectious agents and parasites. Such practices can facilitate the spread of zoonotic diseases, including tuberculosis and trichinellosis, particularly when carcasses are accessible to both wildlife and domestic animals (56–58). Moreover, the persistence of carcasses in the environment may alter local scavenger community dynamics and increase interspecies contact rates, further amplifying the risk of pathogen spillover (59). Addressing illegal disposal therefore requires not only enforcement of existing regulations but also coordinated surveillance and public awareness campaigns to mitigate both ecological and public health consequences.

### Limitations of the study

Although a large number of haplotypes were detected in this study, the major haplotypes, haplotype l-1, haplotype k-2 and haplotype h-2, together accounted for a high percentage, approximately 72%, and this frequency bias may have affected statistical values. A possible reason for this frequency bias could be that the tails submitted to the local government were randomly collected from the same local populations, which may have biased the number of maternally related individuals.

Randomly collected samples may contain many maternally related individuals, and it has been reported that a bias towards maternally related individuals generally reduces the genetic diversity of population samples (60). It is also possible that in small population samples the bias of maternally related individuals may increase the frequency of particularly rare haplotypes (61). The results of the BLAST search showed that most of the haplotypes were novel, with the major haplotypes, haplotype l-1, haplotype k-2 and haplotype h-2, also being novel, suggesting that these may also be rare haplotypes in Japan as a whole. As it is often difficult to collect unrelated specimens in wildlife forensics, this bias towards maternally related individuals must be considered when identifying individuals by mtDNA analysis.

## Data Availability

The datasets presented in this study can be found in online repositories. The names of the repository/repositories and accession number(s) can be found in the article/Supplementary materialS1.
